# Tomato *SlCDF3* Delays Flowering Time by Regulating Different *FT-Like* Genes Under Long-Day and Short-Day Conditions

**DOI:** 10.3389/fpls.2021.650068

**Published:** 2021-05-05

**Authors:** Dawei Xu, Xueou Li, Xue Wu, Lili Meng, Zhirong Zou, Encai Bao, Zhonghua Bian, Kai Cao

**Affiliations:** ^1^Institute of Urban Agriculture, Chinese Academy of Agricultural Sciences, Chengdu, China; ^2^The Agriculture Ministry Key Laboratory of Agricultural Engineering in the Middle and Lower Reaches of Yangtze River, Institute of Agricultural Facilities and Equipment, Jiangsu Academy of Agricultural Sciences, Nanjing, China; ^3^Horticulture College, Northwest A&F University, Xianyang, China; ^4^Department of Plant Science, School of Agriculture and Biology, Shanghai Jiao Tong University, Shanghai, China

**Keywords:** photoperiod, *SlCDF3*, *FT-like* gene, flowering time, tomato

## Abstract

Photoperiod is a crucial inducer of plant flowering. Cycling DOF factors (CDFs) play pivotal roles in the flowering of long-day (LD) and short-day (SD) plants. However, the functions of *CDFs* in the photoperiod regulated flowering remain unclear in day-neutral plants. In the present study, tomato (*Solanum lycopersicum* L. cv. “Ailsa Craig”) seedlings of the wild-type and transgenic lines of overexpressing *CDFs* were treated with different photoperiods. The flowering time and the expression pattern of *SlCDFs* and other *FT-like* genes were investigated. The results showed that tomato *SlCDF1*, *SlCDF2*, *SlCDF3*, *SlCDF4*, and *SlCDF5* are homologs to *Arabidopsis cycling DOF factor 1* (*AtCDF1*). *SlCDF1–5* expression levels were influenced by the developmental stage and the tissue location, and notably, the expression patterns throughout light environments showed two opposite trends. Among the *SlCDF1–5* overexpression transgenic lines, overexpressing *SlCDF3* delayed flowering time in both LD (16 h light/8 h dark) and SD (8 h light/16 h dark) conditions. Furthermore, *SlCDF3* led to an increase in the mRNA level of *SlSP5G*, a tomato *FT-like* gene, in LD conditions, while the transcription level of the other two *FT-like* genes, *SlSP5G2* and *SlSP5G3*, were up-regulated in SD conditions. Taken together, at the transcription level, our results demonstrated that *SlCDF3* played a significant role in controlling tomato flowering under LD and SD conditions, possibly through directly or indirectly regulating *FT-like* genes.

## Introduction

Light is one of the most important factors in plant growth and development, since light is not only the driving force of photosynthesis but acts as an important transduction signal to regulate photomorphogenesis and endogenous substance metabolism via triggering or repressing related gene expressions (Ma et al., 2001). Flowering is a crucial period in plants representing the shift from their vegetative to reproductive phase. For instance, long-day (LD) conditions induced *Arabidopsis* flowering (Andrés and Coupland, 2012), while short-day (SD) conditions promoted early flowering of the *Sorghum* (Wolabu et al., 2016). Although tomato is a typical day-neutral plant (DNP), the flowering time of tomato is delayed under LD conditions and promoted under short day (SD) conditions (Sawhney and Greyson, 1972).

Previous studies reveal that the responses of plant flowering to photoperiod is a very complex process and many key genes are involved in photoperiod-mediated flowering (Kinet, 1977; Song et al., 2015). In *Arabidopsis*, *flowering locus T* (*FT*) encodes florigen, a key protein in triggering flowering, and its transcription is regulated by photoperiods (Kobayashi et al., 1999). Furthermore, *FT* expression is directly and indirectly controlled by a series of upstream transcription factors. Among them, Flavin Kelch Box1 (FKF1), Gigantea (GI), and constans (CO) function as transcriptional activators, whereas short vegetative phase (SVP), flowering loucs C (FLC), tempranillo (TEM), and cycling DOF factor 1 (CDF1) function as transcriptional repressors (Andrés and Coupland, 2012). Phylogenetic tree analysis indicates that CDF proteins belong to a distinct group of DOF transcription factors, the D subfamily. CDF proteins contain a C2–C2 zinc finger at their N-termini, composed of a conserved 51-residue domain, which is a common feature for the DOF family. In addition, CDF proteins have three conserved motifs at their C-termini, which is the basis for their distinctive function (Corrales et al., 2014). There are five *CDF* genes (*AtCDF1–5*) in *Arabidopsis* and overexpression of *AtCDF1* results in flowering delay (Song et al., 2012). Since AtCDF1, the first CDF protein was discovered in plants (Imaizumi et al., 2005), dozens of CDF related genes have been found in other plants, including *Jatropha curcas JcDof1* and *JcDof3* (Yang et al., 2010, 2011) and *Brassica napus BnCDF1* (Xu and Dai, 2016). In the rice, overexpression of *OsDof12* induces high expression of *Heading date 3a* (Hd3a) and early flowering under LD conditions. Conversely, *osdof12* mutants have smaller petals and delay flowering time relative to wild-type plants (Iwamoto et al., 2009; Li et al., 2009). Subsequent studies have revealed that *OsDof4* and *OsDof12* are in the same subfamily, with similar functions in photoperiod-regulated flowering (Wu et al., 2017).

The *in vivo* and *in vitro* experiments have indicated that *AtCDF1* specifically binds with DOF binding elements (DBEs) in the *CO* promoter to inhibit its expression (Imaizumi et al., 2005). And *AtCDF1* could also directly bind to the promoter of *FT* to regulate flowering (Song et al., 2012). However, *AtCDF1* is degraded when integrated with GI-FKF1 protein complexes (Ding et al., 2018). Moreover, Fornara et al. (2009) found that *AtCDF2*, *AtCDF3*, and *AtCDF5* also participated in the regulation of flowering through modulation of the expression of flowering-related genes. Up to date, five *CDF* genes are identified in tomato: *SlCDF1*, *SlCDF2*, *SlCDF3*, *SlCDF4*, and *SlCDF5*. Overexpressed *SlCDF3* but not *SlCDF1* in *Arabidopsis* leads to late flowering through modulation of the expression of flowering-related genes including *CO* and *FT* (Corrales et al., 2014). However, the functions of *SlCDF1–5* in the regulation of tomato flowering are still poorly understood. Our previous studies found three *FT-like* genes (*SlSP5G*, *SlSP5G2*, and *SlSP5G3*) played important roles in the photoperiod-modulated flowering in tomato (Cao et al., 2016, 2018). We hypothesize that there might be a cross-talk between *SlCDF1–5* and *FT-like* in the regulation of tomato flowering. In this study, we generated *SlCDF1–5* overexpression plants and analyzed the transcription levels of downstream *FT-like* genes in LD and SD conditions. Our results showed *SlCDF3* delayed flowering time by regulating different *FT-like* genes, providing a molecular basis for elucidating the regulatory mechanism of flowering in the day-neutral plant.

## Materials and Methods

### Sequence Alignment and Protein Structure Prediction

For our evolutionary analysis, 34 tomato DOF protein sequences reported by Corrales et al. (2014) were downloaded from http://solgenomics.net/ and 36 *Arabidopsis* DOF proteins reported by Lijavetzky et al. (2003) were downloaded from https://www.arabidopsis.org/. We used MegAlign and MEGA 7.0 software to perform amino acid sequence alignment and homology analysis of the target proteins, respectively, and CLUSTAL W to build neighbor-joining trees.

The structures of tomato CDF proteins were analyzed and predicted using the EzMol online tool^1^.

### Plant Material and Growth Conditions

Tomato (*Solanum lycopersicum* L. *var.* Ailsa Craig) seeds were soaked in 50% bleach for 30 min. After sterilization, those seeds were rinsed thoroughly in running water and then incubated at 25°C under dark conditions. After germination, seedlings were sowed into the commercial substrate (PINDSTRUP, Beijing, China) and grown under white LED light (400–700 nm, peaked at 455 and 570 nm) in an environmentally controlled growth chamber with three different photoperiods: LD (16 h light/8 h dark), SD (8 h light/16 h dark), and DN (12 h light/12 h dark). The light intensity, relative humidity, and CO_2_ level in the growth chamber were set at 200 μmol m^–2^ s^–1^, 65%, and 400 μmol mol^–1^, respectively, while the day and night temperature was both set at 25°C.

### RNA and DNA Extraction

To study the spatial expression patterns of SlCDF related genes, we extracted total RNA from the leaf, stem, and root samples of 5-weeks-old plants (three plants per sample and three samples per treatment) grown under DN conditions. Additionally, to study expression changes during development, seven leaves were collected from the top to the bottom of another three 7-weeks-old plants grown under DN conditions. To determine diurnal changes in the expression of *SlCDFs*, the third youngest and fully expanded leaves were collected every 4 h for 24 h (0, 4, 8, 12, 16, 20, and 24 h) from 5-weeks-old plants under SD, LD, and DN conditions. The plant samples were collected as the method described in our previous study (Cao et al., 2016). Total RNA was extracted using an RNeasy Plant mini kit (QIAGEN, Shanghai, China) following the manufacturer’s instructions. Plant genomic DNA was isolated using an Easy Pure Plant Genomic DNA kit (TransGen, Beijing, China) according to the manufacturer’s instructions.

### Gene Isolation, Vector Construction, and Plant Transformation

The ORFs of *SlCDF1* (*Solyc03g115940*), *SlCDF2* (*Solyc05g007880*), *SlCDF3* (*Solyc06g069760*), *SlCDF4* (*Solyc02g067230*), and *SlCDF5* (*Solyc02g088070*) were amplified by PCR from “Ailsa Craig” cultivar cDNAs that had been cloned into a pHELLSGATE8 vector (Invitrogen, Shanghai, China) by recombination using the ClonExpress II One Step Cloning Kit (Vazyme, Nanjing, China). PCR primers required for cloning genes and identifying positive transgenic plants are summarized in Supplementary Table 1. The pHELLSGATE8 vector carries a spectinomycin resistance gene for bacterial selection, a kanamycin resistance gene for the selection of transformed plants, and a CaMV35S promoter. The primers used are listed in Supplementary Table 1. The resulting plasmids were used to transform “Ailsa Craig” tomato plants following the method described by Ellul et al. (2003). The seeds from the transformed plants were harvested and plated onto a selective medium, and the kanamycin-resistant seedlings were transplanted to soil. Transgenic plants were further confirmed by PCR using genomic DNA as a template and amplified with CaMV35S forward and gene-specific reverse primers. Three transgenic lines with high expression were screened by qRT-PCR before the T1 generation; T1 generation seeds were screened by kanamycin and verified by qRT-PCR at the seedling stage before experimental observation.

### Gene Expression and qRT-PCR Analysis

After RNA extraction, cDNA synthesis was performed using the SuperscriptIII First-strand synthesis system (Invitrogen, Shanghai, China) as the manufacturer’s instructions. Real-time PCR was performed using SYBR Premix Ex Taq (Takara, Dalian, China) with a Bio-Rad CFX96 real-time PCR platform (Bio-Rad, Hercules, CA, United States). *GAPDH* transcripts were used as the internal control (Løvdal and Lillo, 2009). The qRT-PCR primers used in this study were listed in Supplementary Table 1. The efficiency of primers was calculated before carrying out the qRT-PCR reaction, all 10 gene primers of good quality with a PCR efficiency (in *r*-squared value) > 0.9 (Supplementary Table 1). The qRT-PCR was performed with three technical replicates.

### Statistical Analysis

All the data were subjected to one-way ANOVA analysis using SPSS 20.0 (SPSS, version 20.0, IBM Inc., Armonk, NY, United States). The significant differences between means were assessed by Duncan’s multiple range test (*P* < 0.05). Error bars in all figures represent standard deviations from the mean.

## Results

### Tomato SlCDF1–5 Were Highly Similar in Amino Acid Sequence and Spatial Structure

**FIGURE 1:**
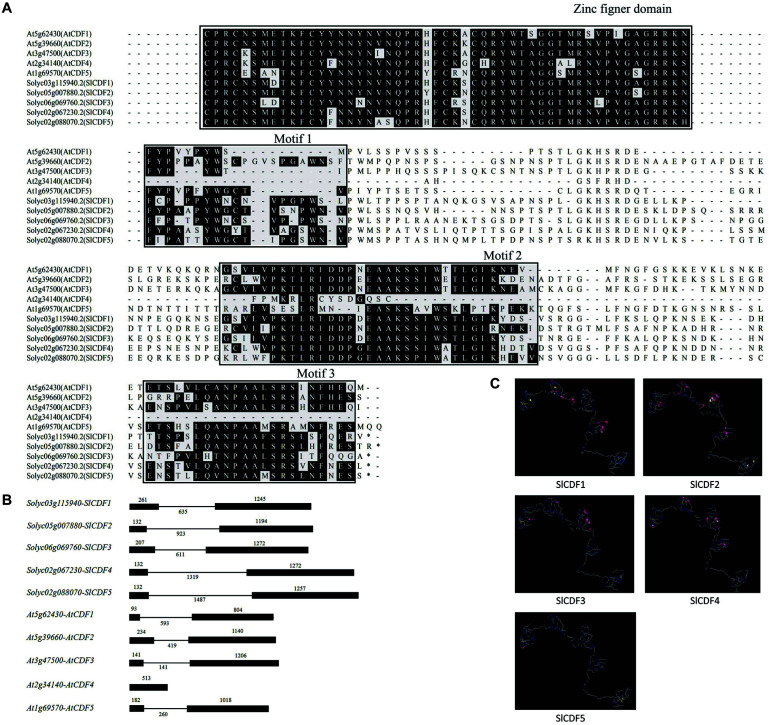
The corresponding amino acid sequences and spatial structures of tomato *SlCDF1–5* genes were highly conserved. **(A)** Amino acid sequence alignment of *Arabidopsis* and tomato CDF proteins. The sequences in the black box were highly conserved. **(B)** Exon-intron structure analysis of *CDF* genes. The black solid boxes represent exons, while the horizontal lines represent introns. **(C)** The simulated three-dimensional structure of tomato SlCDF1–5. The pink and yellow areas represent α-helices and β-sheets, respectively.

We constructed a phylogenetic tree based on the amino acid sequences of 36 *Arabidopsis* DOF and 34 tomato DOF proteins, finding that SlCDF1–5 in tomato were closely related to AtCDF1–5 (Supplementary Figure 1). Simultaneously, sequence alignment results also indicated that, other than AtCDF4, the remaining nine CDF proteins contained C2C2 zinc finger structures at their N-termini and three conserved sequences (21, 33, and 22 residues) at their C-termini (Figure 1A). Notably, AtCDF4 was observed to have only motif 2 at its C-terminus, indicating that it might have a distinct function. In addition to the amino acid sequences, the structures of introns and exons were also highly conserved. At the gene structure, except *AtCDF4*, all other nine *CDF* genes contained two exons and one intron (Figure 1B). The protein spatial conformation in Figure 1C showed that tomato SlCDF1–5 proteins mainly consist of a region of α-helices and β-strands as well as a considerable portion of random structures. Such spatial structures may facilitate CDF proteins binding to the promoters of target genes for transcriptional regulation.

### *SlCDF1–5* Expression Patterns Are Spatially and Temporally Specific

**FIGURE 2:**
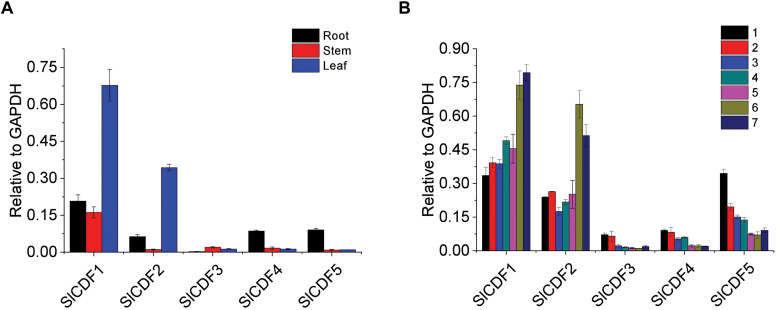
Transcription analyses of tomato *SlCDF1–5* throughout development and among tissues. **(A)** *SlCDF1–5* expression levels in tomato roots, stems, and leaves at 5-weeks-old tomato plants. **(B)** *SlCDF1–5* expression levels in the first seven individual leaves from top to bottom in 7-weeks-old tomato plants. Three technical replicates were performed for each extract, vertical bars on the lines represent the SE (*n* = 3).

The tissue-special expression patterns of related genes often indicate their functions. To investigate the roles of *SlCDF1–5* genes in flowering, we first assayed their expression levels in different vegetative tissues. In total RNA extracted from leaf, stem, and root tissues of 5-weeks-old tomato plants grown under DN conditions, *SlCDF1* and *SlCDF2* were highly expressed in leaves, while *SlCDF4* and *SlCDF5* showed lower expression in roots. The *SlCDF3* expression level was low in all detected tissues (Figure 2A). To further investigate the expression patterns of *SlCDF1–5* across developmental stages, we assayed *SlCDF1–5* expression in true leaves from top to bottom of 7-weeks-old tomato plants grown under DN conditions. *SlCDF1* and *SlCDF2* were highly expressed in all leaves and showed downward trends during development, while *SlCDF3*, *SlCDF4*, and *SlCDF5* showed an opposite trend (Figure 2B).

### *SlCDF1–5* Showed Two Distinct Expression Patterns Under LD and SD Conditions

**FIGURE 3:**
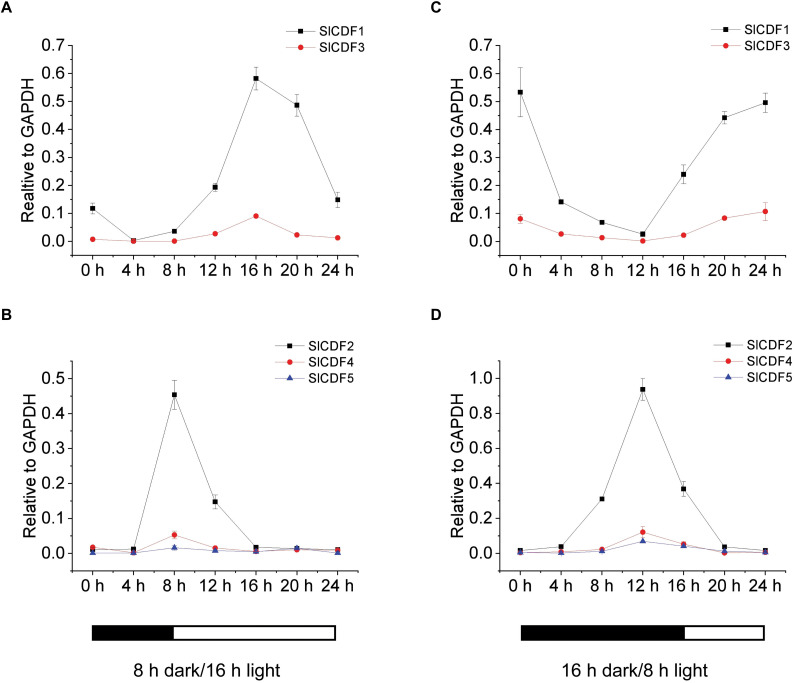
Transcription analyses of tomato *SlCDF1–5* in response to different photoperiods. Expression of *SlCDF1–5* was analyzed by qRT-PCR in 4-weeks-old tomato plants grown under a diurnal cycle of 16 h light/8 h dark **(A,C)** or 8 h light/16 h dark **(B,D)**. White and black bars along the horizontal axis represent light and dark periods, respectively. Three technical replicates were performed for each extract, vertical bars on the lines represent the SE (*n* = 3).

To reveal the relationships between *SlCDF1–5* genes and photoperiod, we examined their diurnal expression patterns under different photoperiod conditions. Based on their expression patterns, *SlCDF1–5* can be categorized into two groups. *SlCDF1* and *SlCDF3* expression peaked after being irradiated for 8 h, and then decreased in the following 8 h under LD conditions (Figure 3A). Under SD conditions, those gene expressions increased from 4 h before the onset of the light period and peaked at the end of the light period (Figure 3B). The other three *SlCDF* genes showed similar expression patterns, with *SlCDF2*, *SlCDF4*, and *SlCDF5* expression peaking at the end of the dark period in LD conditions and at 4 h before the light period under SD conditions (Figures 3C,D). In addition, the same phenomenon was also observed under diurnal-neutral (DN) conditions (Supplementary Figure 2).

### *SlCDF3* Negatively Regulates Flowering by Increasing Transcripts of FT-Like Genes in Both LD or SD Conditions

**FIGURE 4:**
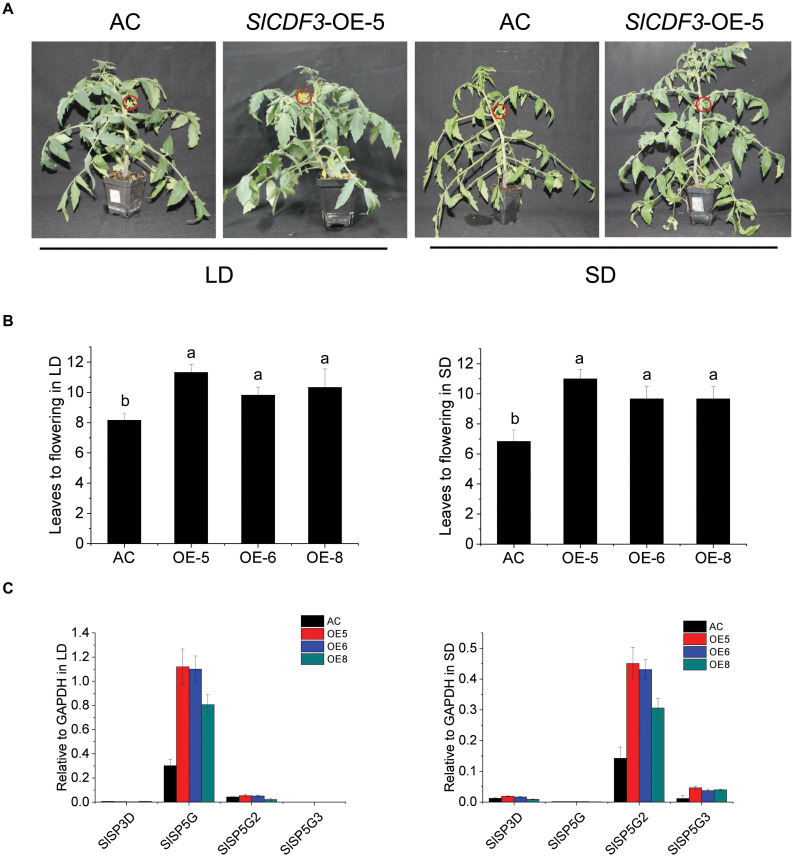
*SlCDF3* delayed flowering by regulating different *FT-like* genes in long-day (LD) and short-day (SD) conditions. **(A)** Flowering phenotypes of *SlCDF3*-overexpressing tomato plants under LD or SD conditions. The red circles indicate flowers. **(B)** Flowering time of *SlCDF3-*overexpressing tomato plants represented by leave number under LD or SD conditions. Vertical bars on the lines represent the SE (*n* = 6). **(C)** The transcription levels of tomato *FT-like* genes in *SlCDF3*-overexpressing tomato plants under LD and SD conditions. Three technical replicates were performed for each extract, vertical bars on the lines represent the SE (*n* = 3).

To explore the mechanism of *SlCDF1–5* in the regulation of flowering under different photoperiods, corresponding overexpression lines were generated. The T0 generation plants were identified by PCR and qRT-PCR, and the three lines with the highest expression levels were selected and transplanted to the greenhouse for cultivation (Supplementary Figure 3). After kanamycin screening, genotyping PCR, and qRT-PCR verifying, T1 generation materials were subjected to different photoperiod conditions. The flowering time of the tomato is calculated as the number of true leaves needed before flowering. Under SD conditions, the number of true leaves of wild-type plants before flowering was eight on average, while the numbers of true leaves before flowering increased to nine under LD conditions (Figure 4A). Overexpression of *SlCDF3* delayed flowering compared to wild-type plants in both LD and SD conditions. The number of leaves before flowering in *SlCDF3-*overexpressing plants (OE-5) increased to 10.7 under SD conditions and to 11.3 under LD conditions (Figure 4B). However, there were no significant differences between the flowering time of other *SlCDFs* overexpression lines and wild-type plants (Supplementary Figure 4).

To investigate the potential interaction between *FT-like* genes and the *SlCDF3* gene, we measured the expression levels of *FT-like* genes in *SlCDF3* overexpressing plants under LD and SD conditions (Figure 4C). Compared with wild-type plants, *SlSP5G* expression in *SlCDF3*-overexpressing plants was significantly increased under LD conditions, while *SlSP5G2* and *SlSP5G3* expression levels were increased under SD conditions. However, there were no distinct changes in the transcription levels of *SlSP5G2* and *SlSP5G3* in LD conditions and the expression level of *SlSP5G* in SD conditions. In addition, to further verify the special interaction between *SlSP5G* and *SlCDF3*, we also studied the expression levels of *SlSP5G* in T1 generation of other four *SlCDFs-*overexpressing tomato plants. These results showed that the transcription levels of *SlSP5G* in *SlCDF1,4,5*-overexpressing tomato plants were not significantly changed, with slightly decreased in *SlCDF2*-overexpressing transgenic lines (Supplementary Figure 5).

## Discussion

Photoperiod is one of the most important environmental factors in the regulation of plant flowering. Tomato is a typical day-neutral plant (DNP), but the flowering time of tomato is also regulated by photoperiod. At the transcription level, our present study demonstrated that *SlCDF3* played an important role in the regulation of tomato flowering via inducing the expression of the downstream *FT-like* genes directly or indirectly.

The CDF proteins belong to the D subfamily of DOF transcription factors. The specific expression pattern of DOF family members has been assessed in many species. The expression levels of *StDof29a*, *StDof32*, and *StDof34* from potato, a congener of tomato, are higher in leaves than other tissues (Li et al., 2009). However, *OsDof11* is predominantly expressed in leaves and promotes flowering in rice by regulating *Hd3a* and *OsMADS14* expression levels under LD conditions (Li et al., 2009). In our present study, the tissue-specific expression of *SlCDF1–5* (Figures 2A,B) indicated *SlCDF1–5* had potentially large differences in biological regulatory functions in tomato plants. According to the study of Corrales et al. (2014), *SlCDF1–5* expression patterns could be divided into two groups under LD and continuous light conditions. In this study, we found that *SlCDF1–5* showed two distinct expression patterns not only in LD conditions but also in DN and SD conditions (Figure 3 and Supplementary Figure 2). These results suggest that the transcription levels of *SlCDF1–5* might be regulated by external light signals and circadian rhythm. Similar results were also reported in other flowering plants, including rice (*Rdd1* and *OsDof4*) (Iwamoto et al., 2009; Li et al., 2009), *Jatropha curcas* (*JcDof1* and *JcDof3*) (Yang et al., 2010, 2011), and potato (Kloosterman et al., 2013).

In most flowering plants, seasonal variation in flowering time largely depends on the expression levels of *FT-like* genes (Laurent and George, 2006; Abelenda et al., 2014). The day length-dependent *FT* expression is governed mainly by the transcriptional activator–CO (Sawa and Kay, 2011). In addition to CO, DOF family members also participate in the regulation of *FT* transcription. For example, AtCDF1, a repressor of CO transcription, controls photoperiodic flowering in *Arabidopsis* (Goralogia et al., 2017). Furthermore, overexpression of rice *OsDof4* leads to earlier flowering under LD conditions, but late flowering under SD conditions (Wu et al., 2017). In our study, the increased transcription level of *SlCDF3* induced delayed flowering in both LD and SD conditions (Figures 4A,B). This is in line with previous results in *Arabidopsis* (Imaizumi et al., 2005). However, except for *SlCDF3*, the increased expression of other *SlCDFs* did not delay the flowering under LD and SD conditions (Supplementary Figure 4). This could partly be explained by the different post-transcriptional regulation patterns or differences in protein stability of SlCDF1–5. Further detailed studies including the post-transcription regulation are still needed to reveal the mechanism of these genes in the regulation of tomato flowering.

Our previous studies found that there are three *FT-like* genes in tomatoes that respond to photoperiod: *SlSP5G*, *SlSP5G2*, and *SlSP5G3* (Cao et al., 2018). Among them, *SlSP5G* is mainly expressed under LD conditions while *SlSP5G2* and *SlSP5G3* were expressed under SD conditions (Cao et al., 2016, 2018). To clarify the main reason for the delayed flowering in *SlCDF3*-overexpressing plants, we measured the expression of *FT-like* genes under LD and SD conditions (Figure 4C). Compared with wild-type plants, *SlSP5G* expression in *SlCDF3*-overexpressing plants was significantly increased under LD conditions, while *SlSP5G2* and *SlSP5G3* expression levels were increased under SD conditions (Figure 4C). Although the *SlCDF3* protein level was not tested with *SlCDF3*-overexpressing plants in the present study, the up-regulation of the *FT-like* genes and the delayed flowering in *SlCDF3*-overexpressing plants suggested the possible regulation of *SlCDF3* on plant flowering via interacting with *FT-like* genes at the transcription level. In LD conditions, *SlCDF3* increases the transcription of *SlSP5G* and resulted late flowering; in SD conditions, *SlCDF3* induces the expression levels of *SlSP5G2* and *SlSP5G3* might be one of the reason for inhibiting tomato flowering. Combined with the little effects of overexpressing *SlCDF3* on the *SlSP5G* expression in SD conditions and the transcriptions of *SlSP5G2* and *SlSP5G3* in LD conditions, these results indicate that other transcription factors might be involved in the regulation of *FT-like* genes in different photoperiod conditions. Although we explained the variation of expression level in *FT-like* genes under LD and SD, whether *SlCDF1–5* directly or indirectly regulates *FT-like* genes remains unknown, and further detailed studies on post-transcriptional modification and protein synthesis are still needed.

## Conclusion

Our present study demonstrated the pivotal role of *SlCDF3* in controlling tomato flowering time at the transcription level and identified the potential cross-talk between *SlCDF3* and *FT-like* genes. *SlCDF3*-overexpressing led to late flowering by inducing transcription of *SlSP5G* under LD conditions, and by triggering the expression of *SlSP5G2* and *SlSP5G3* under SD conditions. These findings could enrich the current understanding of the tomato flowering signal transduction pathway and provide a theoretical basis for related studies on flowering in DNPs.

## Data Availability Statement

The original contributions presented in the study are included in the article/Supplementary Material, further inquiries can be directed to the corresponding author/s.

## Author Contributions

ZB and KC conceived the original research plan and designed the experiment. DX, XL, and XW performed the experiments and analyzed the data. DX and KC wrote the manuscript, while ZB reviewed and edit the manuscript. All authors contributed to the article and approved the submitted version.

## Conflict of Interest

The authors declare that the research was conducted in the absence of any commercial or financial relationships that could be construed as a potential conflict of interest.

## Footnotes

1 http://www.sbg.bio.ic.ac.uk/~ezmol/

## Supplementary Material

Supplementary Figure 1 Phylogenetic analysis of *Arabidopsis* and tomato DOF protein families. The *Arabidopsis* and tomato gene trees were inferred by the neighbor-joining method after alignment of the DOF domains of the 36 *Arabidopsis* and 34 tomato DOF proteins. The resulting Major Clusters of Orthologous Genes (MCOG) A, B, C, and D are indicated. White circles with tables represent *Arabidopsis* CDF proteins, and black circles represent tomato CDF proteins. The numbers nearby tree branches indicate their confidence. The scale bar represents 0.05 estimated amino acid substitutions per site.

Supplementary Figure 2 Transcription analyses of tomato *SlCDF1–5* in response to day-neutral (DN) conditions. *SlCDF1–5* expression levels were assayed by qRT-PCR in 4-weeks-old tomato plants grown under a diurnal cycle of 12 h light/12 h dark. White and black bars along the horizontal axis represent light and dark periods, respectively. Three technical replicates were performed for each extract, vertical bars on the lines represent the SE (*n* = 3).

Supplementary Figure 3 Transcription analyses of target genes in *SlCDF1–5*-overexpressing tomato plants. *SlCDF1* **(A)**, *SlCDF2* **(B)**, *SlCDF3* **(C)**, *SlCDF4* **(D)**, and *SlCDF5* **(E)** expression assayed by qRT-PCR in T0 generation transgenic tomatoes grown under day-neutral (DN) conditions. Three technical replicates were performed for each extract, vertical bars on the lines represent the SE (*n* = 3).

Supplementary Figure 4 Overexpression of *SlCDF1*, *SlCDF2*, *SlCDF4*, and *SlCDF5* had no effect on flowering time of tomato under long day (LD) and short-day (SD) conditions. **(A)** Images of overexpression tomato plants flowering under LD and SD conditions. The red circles indicate flowers. **(B)** Leaf number at flowering in overexpression tomato plants under LD and SD conditions. Vertical bars on the lines represent the SE (*n* = 6).

Supplementary Figure 5 Overexpression of *SlCDF1*, *SlCDF2*, *SlCDF4*, and *SlCDF5* did not change the transcription of *SlSP5G* under long day (LD) conditions. Three technical replicates were performed for each extract, vertical bars on the lines represent the SE (*n* = 3).
