# Supplementary material for: Tomato SlCDF3 Delays Flowering Time by Regulating Different FT-Like Genes Under Long-Day and Short-Day Conditions
Source: Front Plant Sci. 2021 May 5;12:650068. doi: 10.3389/fpls.2021.650068 (PMC8131850; doi:10.3389/fpls.2021.650068)
Supplement: Supplementary file 1 [file Table_1.docx]

| **Supplemental Table 1. Primers used in this study.** | | | |  |
| --- | --- | --- | --- | --- |
| Primer name | Gene ID | Primer sequence | Primer efficiency (r-squared values)* | Experiment |
| SlCDF1-OE-Fw | Solyc03g115940 | TTCATTTGGAGAGGACACGCATGAGGGAAGTGAAGGAACC |  | Constructing the overexpression vectors, the underlined sequence is homologous arm. |
| SlCDF1-OE-Rv | Solyc03g115940 | ATCTCATTAAAGCAGGACTCTTATACTCTCTCCTGGAAGC |  |  |
| SlCDF2-OE-Fw | Solyc05g007880 | TTCATTTGGAGAGGACACGCATGTCTGAAGTTAGAGATCCT |  |  |
| SlCDF2-OE-Rv | Solyc05g007880 | ATCTCATTAAAGCAGGACTCTCATCGTGTACTCTCACGAA |  |  |
| SlCDF3-OE-Fw | Solyc06g069760 | TTCATTTGGAGAGGACACGCATGACTTGTGATTCAGAGATC |  |  |
| SlCDF3-OE-Rv | Solyc06g069760 | ATCTCATTAAAGCAGGACTCTTAGGCACCTTGTTGGAAGG |  |  |
| SlCDF4-OE-Fw | Solyc02g067230 | TTCATTTGGAGAGGACACGCATGACAGACCCCGCAATTAA |  |  |
| SlCDF4-OE-Rv | Solyc02g067230 | ATCTCATTAAAGCAGGACTCTTATAAGCTCTCATTGAAATT |  |  |
| SlCDF5-OE-Fw | Solyc02g088070 | TTCATTTGGAGAGGACACGCATGTCTGAAGCAATTGCTAT |  |  |
| SlCDF5-OE-Rv | Solyc02g088070 | ATCTCATTAAAGCAGGACTCTTATGAGCTCTCATTGAAAT |  |  |
| CaMV35S-Fw |  | ACGCACAATCCCACTATCCTTC |  | Detecting for transgenic positive tomato plants |
| SlCDF1-q-Fw | Solyc03g115940 | TCATGATTTCTGAAGCCCTT | 0.91 | qRT-PCR |
| SlCDF1-q-Rv | Solyc03g115940 | TCCATTGGGTTTGAACACTG |  |  |
| SlCDF2-q-Fw | Solyc05g007880 | GCTGAGAATTCACATAATATGC | 0.98 |  |
| SlCDF2-q-Rv | Solyc05g007880 | GATTGATCATTCCCGATTGT |  |  |
| SlCDF3-q-Fw | Solyc06g069760 | ATCTCCAAATGGGTTAATTC | 0.94 |  |
| SlCDF3-q-Rv | Solyc06g069760 | ATAGGTTCACACAGGGGTA |  |  |
| SlCDF4-q-Fw | Solyc02g067230 | AACAATGCATAATTGCTCAC | 0.95 |  |
| SlCDF4-q-Rv | Solyc02g067230 | CATTATCTCCAACTACGTAA |  |  |
| SlCDF5-q-Fw | Solyc02g088070 | CTCCTTGGCCATACATATGG | 0.96 |  |
| SlCDF5-q-Rv | Solyc02g088070 | GACATTAGCCTTGAGACCTTA |  |  |
| SP3D-q-Fw | Solyc03g063100 | CCTAGAGAACGTGATCCTCT | 0.99 |  |
| SP3D-q-Rv | Solyc03g063100 | CTTAGGCCAATAGTTCTTGT |  |  |
| SP5G-q-Fw | Solyc05g053850 | CTAGAGATCCTTTAATAGTTTCTGG | 0.99 |  |
| SP5G-q-Rv | Solyc05g053850 | AACCACACCAAAGTCTACAC |  |  |
| SP5G2-q-Fw | Solyc11g008640 | TGCTACAACTTGCAAATTGAT | 0.94 |  |
| SP5G2-q-Rv | Solyc11g008640 | TTCCTCCGACCTTTAGCCTT |  |  |
| SP5G3-q-Fw | Solyc11g008650 | TCTCTTTATTTCGTCAATTGGG | 0.99 |  |
| SP5G3-q-Rv | Solyc11g008650 | AGCAAATTGCCTTGTATTGA |  |  |
| GAPDH-Fw | Solyc03g111010 | ACCACAAATTGCCTTGCTCCCTTG | 0.96 |  |
| GAPDH-Rv | Solyc03g111010 | ATCAACGGTCTTCTGAGTGGCTGT |  |  |

*To calculate qRT-PCR primer efficiency (E), use formula E = (1 + r-squared value).
