# Supplementary figures and images for: Tomato SlCDF3 Delays Flowering Time by Regulating Different FT-Like Genes Under Long-Day and Short-Day Conditions
Source: Front Plant Sci. 2021 May 5;12:650068. doi: 10.3389/fpls.2021.650068 (PMC8131850; doi:10.3389/fpls.2021.650068)

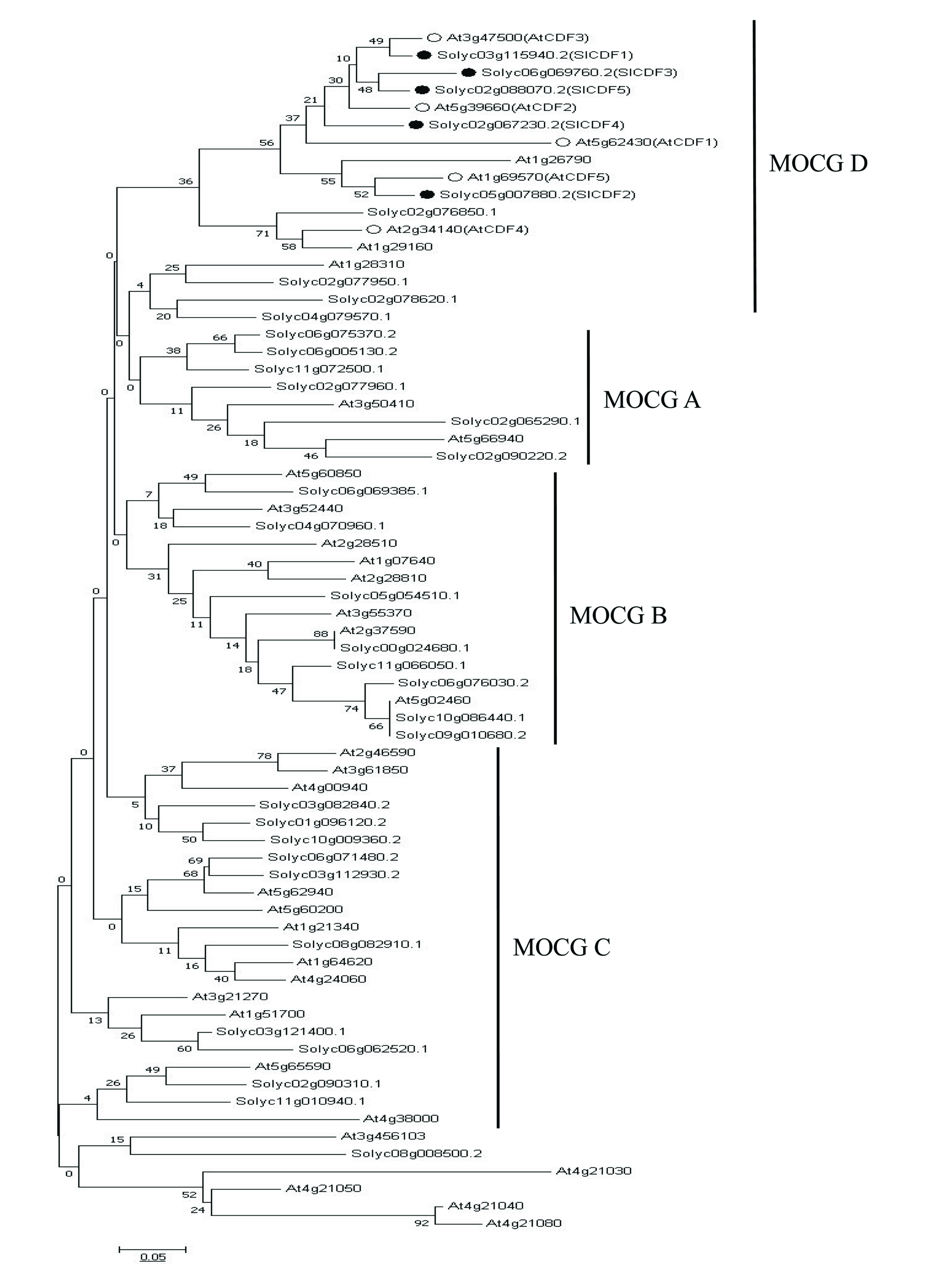

Supplement: Supplementary Figure 1 — Phylogenetic analysis of Arabidopsis and tomato DOF protein families. The Arabidopsis and tomato gene trees were inferred by the neighbor-joining method after alignment of the DOF domains of the 36 Arabidopsis and 34 tomato DOF proteins. The resulting Major Clusters of Orthologous Genes (MCOG) A, B, C, and D are indicated. White circles with tables represent Arabidopsis CDF proteins, and black circles represent tomato CDF proteins. The numbers nearby tree branches indicate their confidence. The scale bar represents 0.05 estimated amino acid substitutions per site. [file Image_1.JPEG]

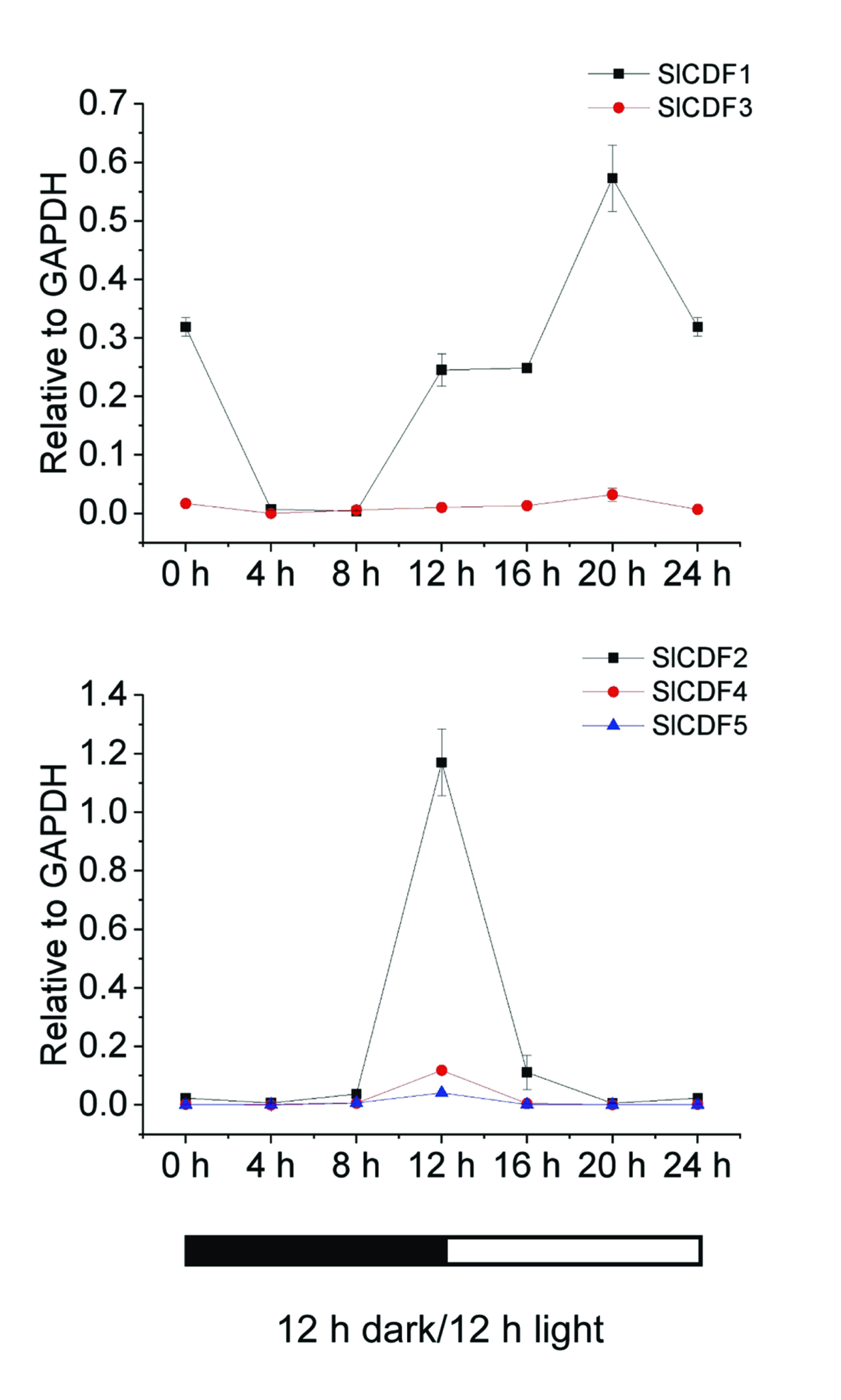

Supplement: Supplementary Figure 2 — Transcription analyses of tomato SlCDF1–5 in response to day-neutral (DN) conditions. SlCDF1–5 expression levels were assayed by qRT-PCR in 4-weeks-old tomato plants grown under a diurnal cycle of 12 h light/12 h dark. White and black bars along the horizontal axis represent light and dark periods, respectively. Three technical replicates were performed for each extract, vertical bars on the lines represent the SE (n = 3). [file Image_2.JPEG]

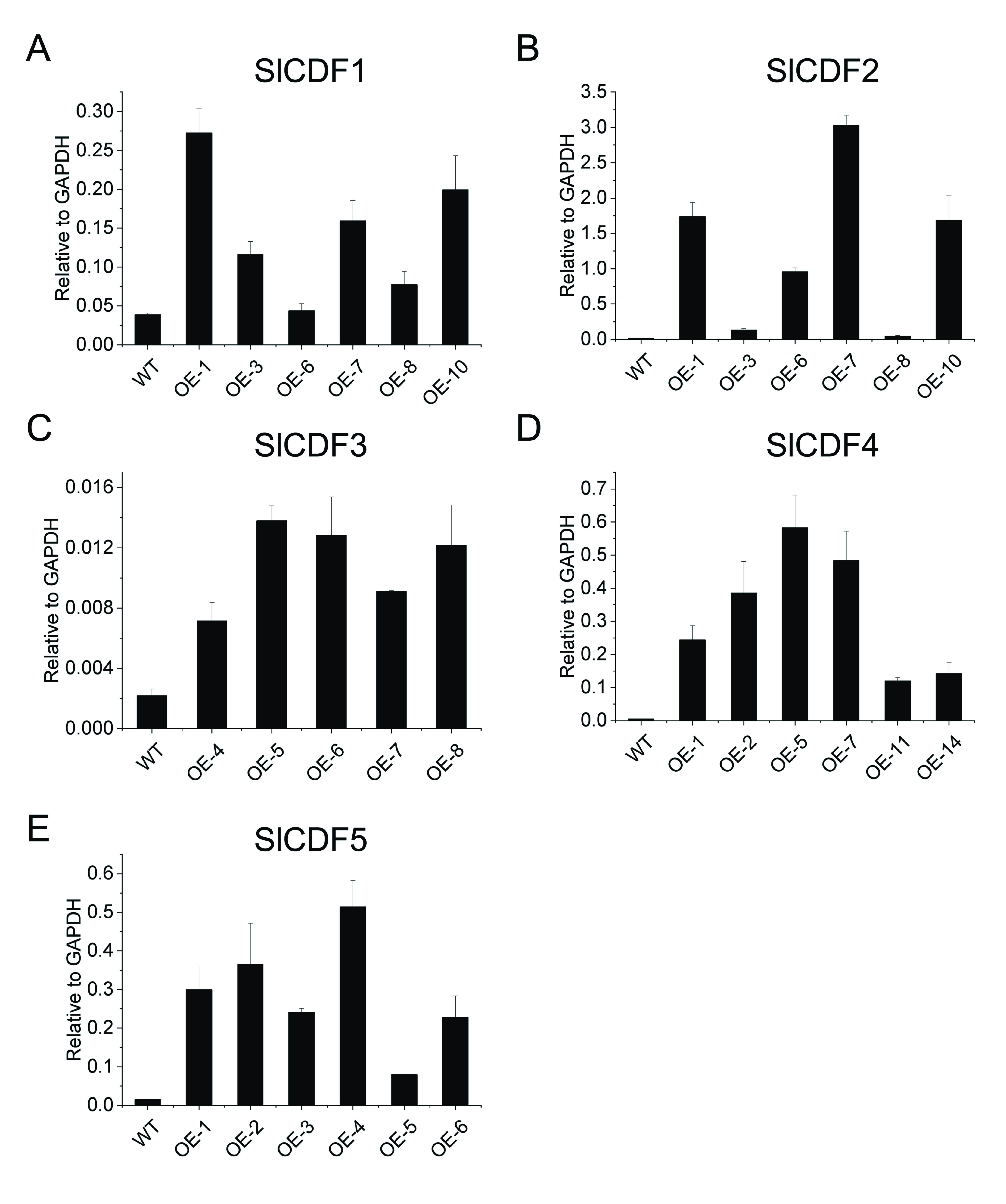

Supplement: Supplementary Figure 3 — Transcription analyses of target genes in SlCDF1–5-overexpressing tomato plants. SlCDF1 (A), SlCDF2 (B), SlCDF3 (C), SlCDF4 (D), and SlCDF5 (E) expression assayed by qRT-PCR in T0 generation transgenic tomatoes grown under day-neutral (DN) conditions. Three technical replicates were performed for each extract, vertical bars on the lines represent the SE (n = 3). [file Image_3.JPEG]

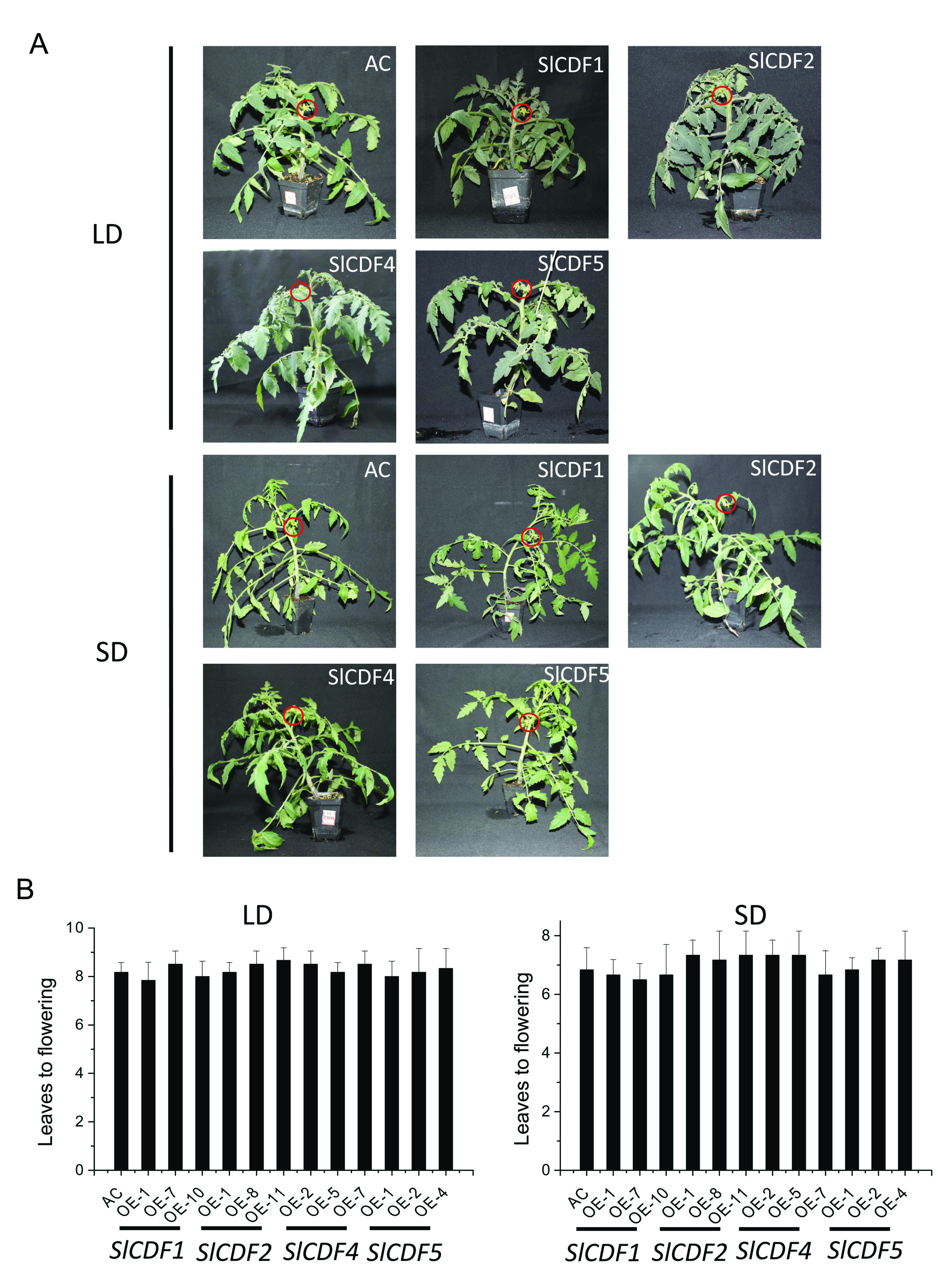

Supplement: Supplementary Figure 4 — Overexpression of SlCDF1, SlCDF2, SlCDF4, and SlCDF5 had no effect on flowering time of tomato under long day (LD) and short-day (SD) conditions. (A) Images of overexpression tomato plants flowering under LD and SD conditions. The red circles indicate flowers. (B) Leaf number at flowering in overexpression tomato plants under LD and SD conditions. Vertical bars on the lines represent the SE (n = 6). [file Image_4.JPEG]

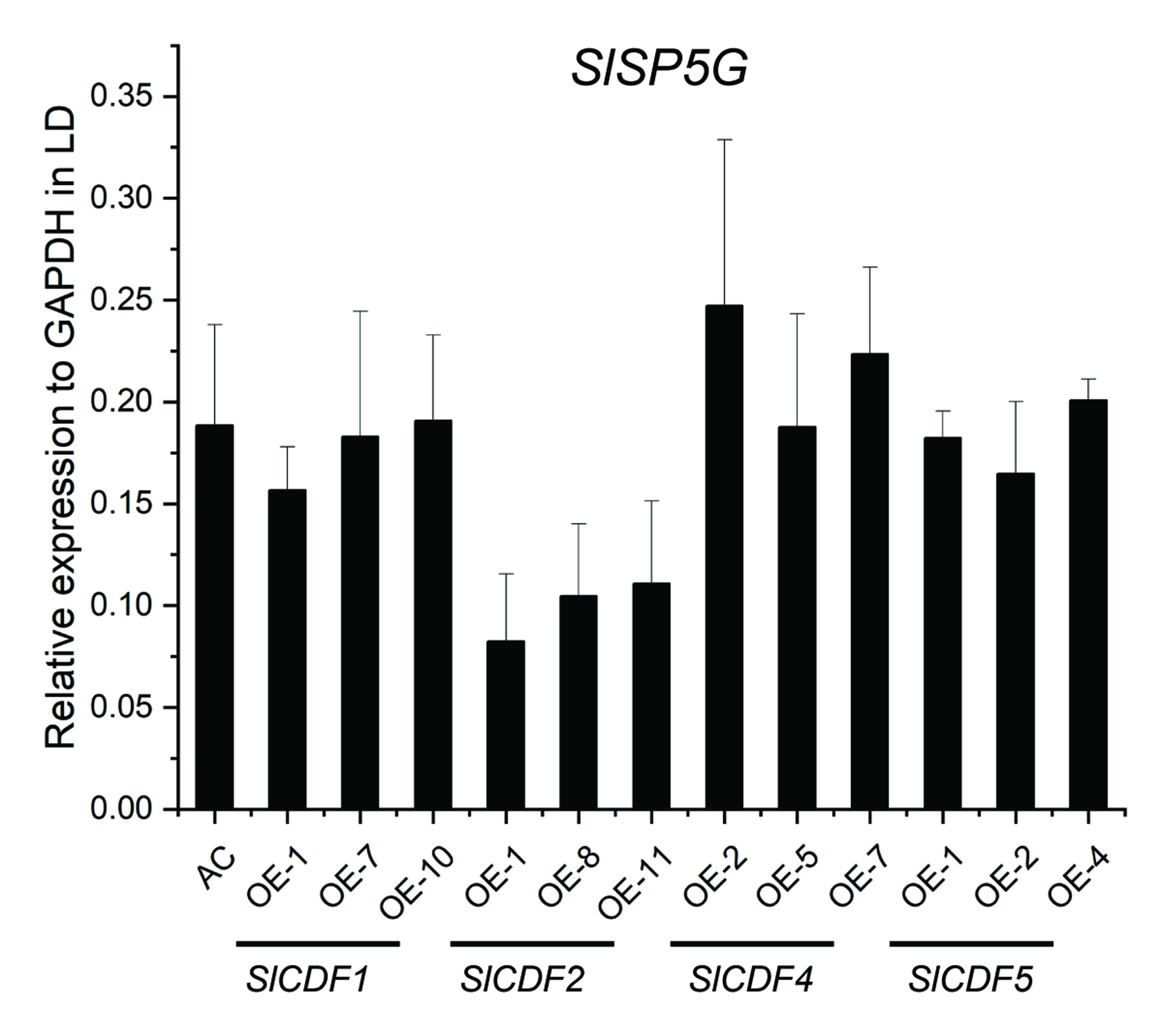

Supplement: Supplementary Figure 5 — Overexpression of SlCDF1, SlCDF2, SlCDF4, and SlCDF5 did not change the transcription of SlSP5G under long day (LD) conditions. Three technical replicates were performed for each extract, vertical bars on the lines represent the SE (n = 3). [file Image_5.JPEG]
